# A model for measuring healthcare accessibility using the behavior of demand: a conditional logit model-based floating catchment area method

**DOI:** 10.1186/s12913-021-06654-3

**Published:** 2021-07-06

**Authors:** Hoon Jang

**Affiliations:** College of Global Business, Korea University Sejong Campus, 2511 Sejong-ro, Sejong, Republic of Korea

**Keywords:** Access to health, Floating catchment area, Conditional logit, Realistic preference, Obstetric care service

## Abstract

**Background:**

Estimating realistic access to health services is essential for designing support policies for healthcare delivery systems. Many studies have proposed a metric to calculate accessibility. However, patients’ realistic willingness to use a hospital was not explicitly considered. This study aims to derive a new type of potential accessibility that incorporates a patient’s realistic preference in selecting a hospital.

**Methods:**

This study proposes a floating catchment area (FCA)-type metric combined with a discrete choice model. Specifically, a new FCA-type metric (clmFCA) was proposed using a conditional logit model. Such a model estimates patients’ realistic willingness to use health services. The proposed metric was then applied to calculate the accessibility of obstetric care services in Korea.

**Results:**

The clmFCA takes advantage of patients’ realistic preferences. Specifically, it can represent each patient’s heterogeneous characteristics regarding hospital choice. Such characteristics include bypassing behavior, which could not be considered using prior FCA metrics. Empirical analysis reveals that the clmFCA avoids the misestimation of accessibility to health services to an extent.

**Conclusions:**

The clmFCA offers a new framework that more realistically estimates patients’ accessibility to health services. This is achieved by accurately estimating the potential demand for a service. The proposed method’s effectiveness was verified through a case study using nationwide data.

**Supplementary Information:**

The online version contains supplementary material available at 10.1186/s12913-021-06654-3.

## Background

Identifying access to health services has been recognized as an important issue for policymakers and researchers in the public health domain. Many countries, including the United States, have put great effort into measuring access to health services. Measuring such access is particularly important for populations living in rural areas, which are more likely to suffer from limited access to such services. It is important to note that if health service suppliers are not properly distributed, it may result in increased unsatisfied demand and lead to undesirable health outcomes among patients. Therefore, accurately measuring access to health services is key to improving overall access to health services and minimizing health disparities [1, 2].

What is access to health services? In crude terms, it can be defined as the ease of using health services to achieve a desired health level. It is also defined as “the outcome of a process, determined by an interplay between the characteristics of the healthcare service system and potential users” [3]. The seminal work by Penchansky and Thomas [4] proposed access process components using five dimensions: availability, accessibility, accommodation, affordability, and acceptability. Although this view has multi-dimensional characteristics, many other researchers have focused on distinguishing potential and realized (or revealed) accessibility [2, 5, 6]. Among them, it is worth noting that Guagliardo [2] indicated that Penchansky and Thomas’ five characteristics could be regarded as the barriers that “impede progression from potential to realized access.”

Realized (or revealed) accessibility refers to the actual use of health services. It is calculated based on the actual utilization of health services [2, 5, 7–9]. Generally, obtaining a value for realized accessibility may be challenging because it requires tremendous costs and resources such as a systemized data collection infrastructure. On the other hand, potential accessibility considers the “probable utilization of health services” [10]. It is generally defined as the supply of health services to a nearby needy population. Measuring potential accessibility as realistically as possible is of persistent interest to health providers or health policymakers. This is because monitoring the current *status quo* of health service provision is important in designing better policies and planning for better health services.

One of the notable metrics of potential accessibility is the floating catchment area (FCA). This metric was originally derived from the gravity-based measure proposed in Weibull’s study [11]. By adapting the gravity-based formula, it simultaneously considers the distance between demand and supply and the supplier’s capacity. Following the publication of this work, subsequent researchers have studied the FCA method. The most famous version of the method is the two-step FCA (2SFCA) [6]. Using population and supply level information, this method calculates the ratio of supply (i.e., physicians) to the population. Although the 2SFCA has effectively measured accessibility in many areas, it crucially failed to address some realistic features, such as potential competition among the population. This is because it equally allocates demand to hospitals without considering the other nearby hospitals’ availability. This results in an unreliable access score for decision-makers. Owing to its simplicity, it looks to further extensions such as the enhanced 2SFCA, 3SFCA, and M2SFCA [12–14].

For instance, the 3SFCA was developed to overcome the shortcomings of the 2SFCA. Introducing a competition weight based on travel time calculates a reasonable amount of demand, thereby reducing the risk of demand overestimation. Similarly, the M2SFCA proposed by Delamater [14] comes from the motivation that all prior metrics have the same underlying assumption. That is, all suppliers’ locations are optimally configured to meet the need regarding health services. To address this, Delamater [14] introduced a decaying function for both demand and supply. In doing so, M2SFCA offers more realistic access levels than other existing models.

Recently, researchers have studied various extensions and applications of the developed FCA methods. For instance, new FCA-type metrics were proposed by considering various transportation modes [15–17] or non-spatial factors, such as gender, income level, etc. [18–22].

Although prior works regarding the FCA have been extensively studied, there have been no attempts to explicitly consider the heterogeneous characteristics of demand for the computation of FCA metrics. Specifically, all existing metrics primarily assume that individuals have a single homogenous preference when using health services. Therefore, regardless of the situation in reality, existing FCA metrics equally divide the demand into two reachable suppliers with equal service capacities. Additionally, the prior metrics consider an interaction between demand and supply only by a weight function, defined by distance or travel time. A weight function is typically defined as a gradually decreasing function by distance (or time). As such, prior metrics only have a diminishing mechanism in calculating accessibility to health services. However, there may be cases in which patients prefer to visit hospitals located far away over hospitals near their residence. This is another limitation of the prior FCA metrics.

Motivated by this, the present study proposes a new FCA metric by incorporating the heterogeneous characteristics of demand. As noted above, while prior metrics are effective to some extent, they do not elucidate the complex nature of access to health services in real-world settings. Specifically, they do not reflect each individual’s unique priorities when choosing their healthcare provider. This study used a discrete choice model to address this issue. The discrete choice model, which has been extensively studied in economics, provides a rigorous mathematical formula to calculate the patient’s willingness to use a certain healthcare provider. Thus, it provides a more realistic representation of individuals’ preferences when choosing a health provider. The proposed metric begins by using a conditional logit model to estimate patients’ willingness to use health services. The author believes that this increases the accuracy of calculating the potential demand for a specific healthcare provider. Consequently, this ultimately provides a more accurate accessibility score. Considering that obtaining the true realized accessibility requires significant effort, this study offers a unique position in calculating more realistic potential accessibility by using a discrete choice model.

Thereafter, the conditional logit model-based FCA (clmFCA) was applied to a real-world setting to examine the differences between the prior metrics and the proposed metric. The clmFCA was used to calculate the accessibility of the obstetric care system in Korea. Just as many countries encounter difficulties in providing adequate levels of obstetric care services in rural areas, Korea also currently suffers from its obstetric care system’s poor level in rural areas [23–25]. This is mainly due to such reasons as a low birth rate and the increasing financial burden incurred in sustainably operating hospitals. Improving accessibility to obstetric care systems is an urgent and vital task for the Korean government because such problems may ultimately lead to many medical and socio-economic problems [26].

In this regard, this empirical analysis contributes to a better understanding of obstetric patients’ accessibility to hospitals across the country. Through a comparison with traditional measures, this study determines how the differences among accessibility measures manifest in real-world settings.

## Methods

### Discrete choice model

To incorporate the realistic behavior of potential demand, a discrete choice model that is based on the random utility theory is applied. This concept was originally introduced as a stimulus in psychology and economics [27, 28]. Among many different choice models, this study uses the conditional logit model (CLM), assuming that the errors are independent and identically distributed through a Gumbel distribution. Notably, McFadden [29] first introduced the CLM, which has been extensively used in many areas, including economics, marketing, and operations management, because of its simplicity and tractability.^1^

The implementation of the clmFCA requires describing the probability that a patient *i* will select hospital *j*, *P*_*ij*_. For this purpose, this study adopts the results presented in the work of Hwang et al. [30], who used the same experimental data as in the present study.

Hwang et al. [30] analyzed patient preferences using CLM. They first reviewed the literature to identify the factors that contribute to the utility function. In this study, obtaining personal data and private information is not permitted because of privacy issues. This information is not easily obtained because demographic data, residential location information, and hospital visit records may identify specific individuals. Therefore, we used the following four covariates shown in Table 1. Details including the collection of studied data and variable selection procedure are provided in the Supplemental Information.

**Table 1:** Covariates applied for estimating patients’ preference

Covariate	Definition	Type (Level)
*LvH*	Level of hospital	Categorical (High (tier 3)/Low (tier 1, tier 2))
*Urb*	Degree of urbanization at hospital *j*	Categorical (Metro/City/Rural)
*Num*	Number of obstetric specialists	Integer (Persons)
*τ*	Travel time by ground transportation unit	Integer (Minutes)

Using the dataset, Hwang et al. [30] subsequently developed the utility function *V*_*ij*_ using eq. (1)^2^:

1 $$ {V}_{ij}=-1.072{LvH}_{High}^j-0.174{Urb}_{City}^j-0.862{Urb}_{Rural}^j+0.151{Num}_j-0.064{\tau}_{ij} $$

In eq. 1, each coefficient represents the relative likelihood of selecting a healthcare provider. For instance, − 1.072 for *LvH*^*j*^_*High*_ is the probability of choosing a higher-level hospital (tertiary/tier 3), which is 0.34 times higher than the probability of selecting a lower-level hospital (primary/tier 1 or secondary/tier 2) (exp (− 1.072) = 0.34). In summary, their model indicates that patients prefer to use the following: 1) lower-tier hospitals, 2) hospitals located in metropolitan (or urban) areas, 3) hospitals with more obstetrics specialists, and 4) hospitals located closer to patients’ residential locations. Using multiple validating procedures, Hwang et al. [30] confirmed that this model could estimate a patient’s preference for choosing a hospital. Details of the validation process, including statistical results, are provided in the Supplemental Information.

### The conditional logit model-based floating catchment area method

Prior FCA metrics still do not address the issue of estimating the true demand for health services despite that they have been widely applied to many areas. As mentioned earlier, a well-estimated demand for the calculation of spatial accessibility plays a key role in measuring true accessibility to health services. It helps hedge the risk of underestimating (or overestimating) the potential volume of demand for services, ultimately leading to designing more effective support programs and policies.

Motivated by this, the present study proposes a metric that incorporates a discrete choice model. It mathematically computes an individual’s willingness to use a specific healthcare provider to more accurately calculate the potential demand for services. Specifically, a conditional logit model attempts to create a decision mechanism of how an individual chooses his or her favored alternatives among the available options. For instance, the choice of which product to buy or which transportation mode to use to go to work are typical examples that can be analyzed by discrete choice models.

Based on a more realistic approximation of how an individual chooses their hospital, a new FCA metric is proposed in Table 2. Such a metric can consider realistic information regarding an individual’s hospital preference.

**Table 2:** The algorithm for calculating the proposed metric

Input{*P*_*ij*_ for *i* ∈ I, *j* ∈ J, *S*_*j*_ for *j* ∈ J, *D*_*i*_ for *i* ∈ I} = Output{*A*_*i*_, for *i* ∈ I}	
**Step 1**. Calculate the choice probability, *P*_*ij*_ for each demand–supply pair (*i*, *j*)	
- ${P}_{ij}=\frac{\exp \left({V}_{ij}\right)}{\sum_{k\in J}\exp \left({V}_{ik}\right)}$	(2)
**Step 2**. Calculate the supply to potential demand ratio, *R*_*ij*_, by fixing each facility *j*	
- ${R}_{ij}=\frac{S_j}{\sum_{i\in I}{D}_i{P}_{ij}}$	(3)
**Step 3**. Sum the ratio, *R*_*ij*_*,* for each population location *i*	
- ${A}_i={\sum}_{j\in {J}_i}{R}_{ij}{P}_{ij}$	(4)

It must be noted that *S*_*j*_ is the capacity of hospital *j*, and *D*_*i*_ denotes the demand (i.e., the number of potential patients for health services) at *i*. The clmFCA consists of three stages, beginning with the calculation of the choice probability of the demand. In step 1, the clmFCA computes the choice probability for each pair (*i*, *j*) using a discrete choice model. Here, it is assumed that the entire population located in *i* has the same preference for choosing hospitals.^3^ The factors (*V*_*ij*_) and data for computing probability should be selected beforehand and prepared accordingly. Next, the clmFCA calculates the supply to potential demand ratio for each facility *j*. It directly applies the selection probability from *i* to *j* rather than using a travel-like impedance function as is found in classical FCA methods. This is a unique characteristic of the proposed metric. Specifically, it considers willingness to use a hospital as part of demand, which is combined with the choice probability (*D*_*j*_*P*_*ij*_) of the demand. This is important in the sense that the clmFCA can consider features that occur in the real world. Finally, the clmFCA adds the calculated values obtained from step 2 with the choice probability for each location *i*.

Overall, the proposed method can systematically consider a user’s preference in selecting health providers, unlike the conventional FCA methods. Particularly, the proposed method can account for both the user’s bypass behavior, that is, when a patient bypasses the nearest hospital and prefers using a far-away one, and the population’s heterogeneous nature. In the virtual experiments, we confirmed that the proposed method is better than some conventional FCA methods when considering a patient’s realistic characteristics (see Supplemental Information for more details). This will help readers understand the difference between the proposed measure and the benchmarking methods.

## Case study: analyzing the potential accessibility of South Korea’s obstetrics care

### Data

A case study using real data was conducted to examine the proposed model’s effectiveness. This study was approved by the Institutional Review Board of the National Medical Center in Korea (IRB No. H-1604-065-003). The data were anonymized in regard to identifying information. Thus, informed consent was not required.

**Fig. 1:**
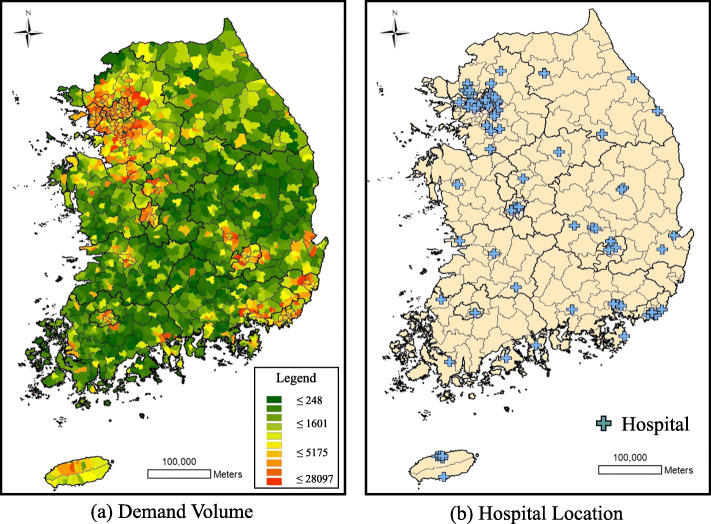
Potential demand for health service (**a**); Hospital location (**b**)

This study used two types of data. One is the demand-side data from the fertile age group (15–49 years old) in Korea, gathered from Korea’s National Statistical Office. As of 2015, the total number of fertile women in Korea was approximately 13 million. Most of them were living in metropolitan areas such as Seoul. For patient location, the present study uses the weighted centroids of each sub-municipal areal unit and not individual residential locations. As of 2015, there were 3488 sub-municipal areal units in Korea. Moreover, this study uses the smallest available areal unit for analysis because it is extremely difficult to access each individual residential address for privacy and confidentiality reasons. Fig. 1(a) shows the geographical distribution and potential demand for services (i.e., the potential number of patients who may use health services).

For the supply side data, we used hospital information obtained from the Health Insurance Review and Assessment Service (HIRA). As of 2015, there were 576 hospitals performing at least one labor and delivery service. Specifically, the capacity (beds) and geographical location information were obtained from the HIRA dataset. It should be noted that Korea operates a three-tiered healthcare delivery system, where tier 3 provides the highest level of medical services. Figure 1(b) shows the hospitals’ locations across the country. Similar to the distribution of demand, most hospitals are located in densely populated areas (e.g., the northwest region).

The last piece of data is travel time. This is obtained based on the national road network provided by the National Transportation Database Service in Korea. Using the ArcGIS™^10.0^ network analysis tool, this study calculated the travel time of all pairs between the residential locations of demand (centroid of the municipal areal unit) and hospital locations. Based on the results of the study conducted by the Korean government, the threshold of whether a patient is within a catchment area of a particular healthcare provider *j* is set as 60 min.^4^

### Results

#### Accessibility scores

Three well-known benchmarking accessibility measures were chosen (2SFCA, 3SFCA, and M2SFCA) to compare the accessibility scores of the clmFCA. It would be beneficial to discuss the advantages and disadvantages of the proposed method because the chosen measures have different characteristics. A short illustration of these measures is presented in the Supplemental Information.

Table 3 presents the basic descriptive statistics for accessibility outcomes. The clmFCA provides the lowest average value of accessibility when compared to the three benchmarking measures. The average access to healthcare provider sets was 0.00098. The highest values were obtained for the 2SFCA (0.000183). However, the standard deviation of accessibility scores from the clmFCA showed the highest values, while the 2SFCA showed the lowest.

**Table 3:** Descriptive statistics for each FCA metric (10^−4^)

Metric	Average	Standard deviation	Minimum	Maximum
2SFCA	1.83	0.74	0.00	4.44
3SFCA	1.67	1.26	0.00	19.5
M2SFCA	1.02	0.97	0.00	17.2
clmFCA	0.98	1.27	0.00	4.38

**Fig. 2:**
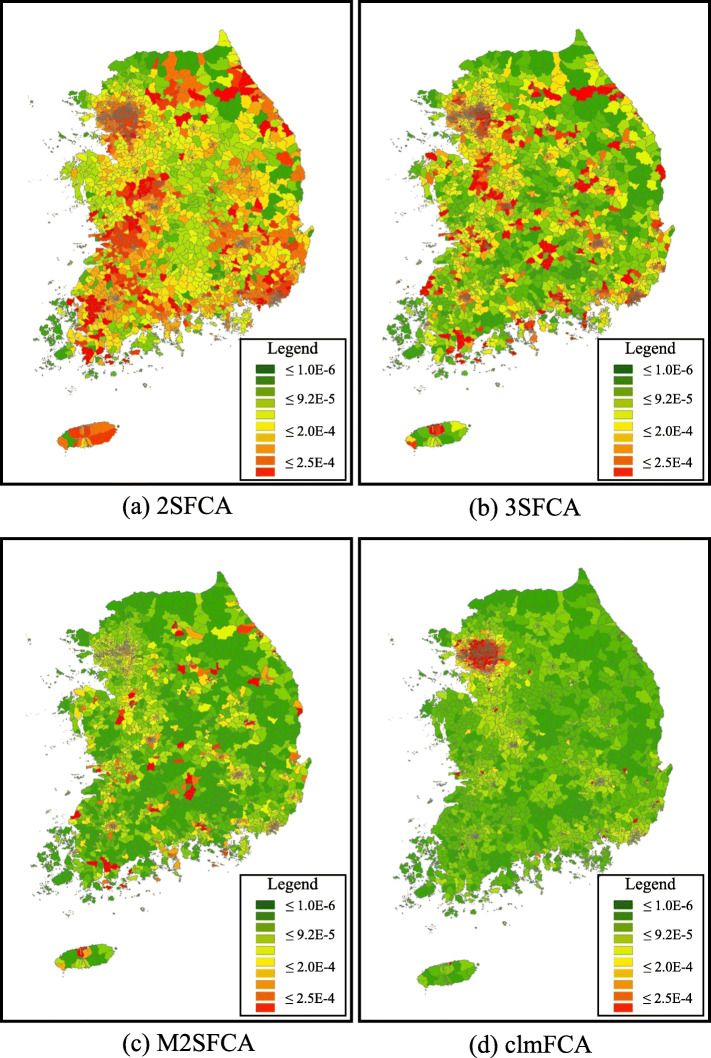
Accessibility scores of (**a**) 2SFCA; (**b**) 3SFCA; (**c**) M2SFCA; (**d**) clmFCA

Next, accessibility maps of the four metrics were presented. Figures 2(a) to (d) show the access scores of healthcare providers. As seen in Fig. 2, the geographical patterns of all four metrics were generally similar. Most regions in green are generally rural areas. Those in yellow and orange are urban and metropolitan areas, respectively.

**Fig. 3:**
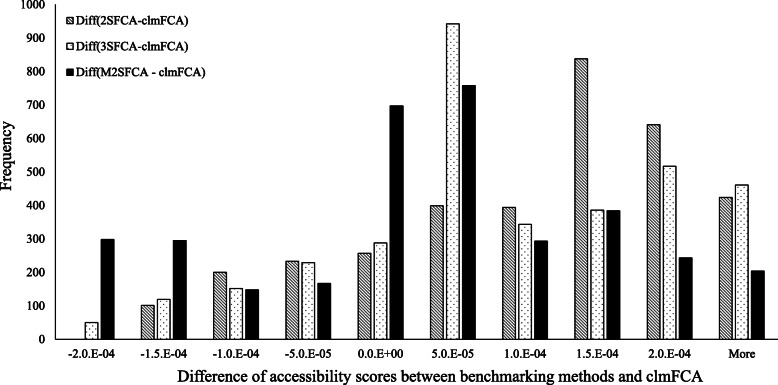
Histogram of differences in accessibility score between prior metrics and the clmFCA

Although the geographical patterns of all four measures are analogous, the magnitude of accessibility is still different. Consistent with the results from Table 3, the clmFCA showed the lowest level of access to healthcare providers, compared to other metrics. Figure 3 presents the degree of difference between the clmFCA and the benchmarking measures to more clearly understand this difference. In the case of 2SFCA, 2793 regions (over 3487 sub-municipal areal units) showed higher accessibility scores than the clmFCA. Although the degree of difference is different, it can be similarly observed that the benchmarking methods provide a higher accessibility score than the clmFCA.

#### Comparison with real observations

Four accessibility scores were compared with a timely relevance index (TRI). The TRI is a primary measure of real accessibility used in Korea to examine how potential accessibility computed from the four metrics is similar to real observations. TRI is defined as the proportion of hospital usage within a time threshold (e.g., 60 min) over the total number of instances of hospital usage in a particular areal unit (e.g., municipal areal unit). A high value indicates that more people use health providers that can be reached within an hour. This is based on the actual use of health services. Moreover, it considers the geographical proximity between demand and supply (health provider). Therefore, the TRI is sufficiently regarded as revealed accessibility, considering geographical proximity.^5^

**Fig. 4:**
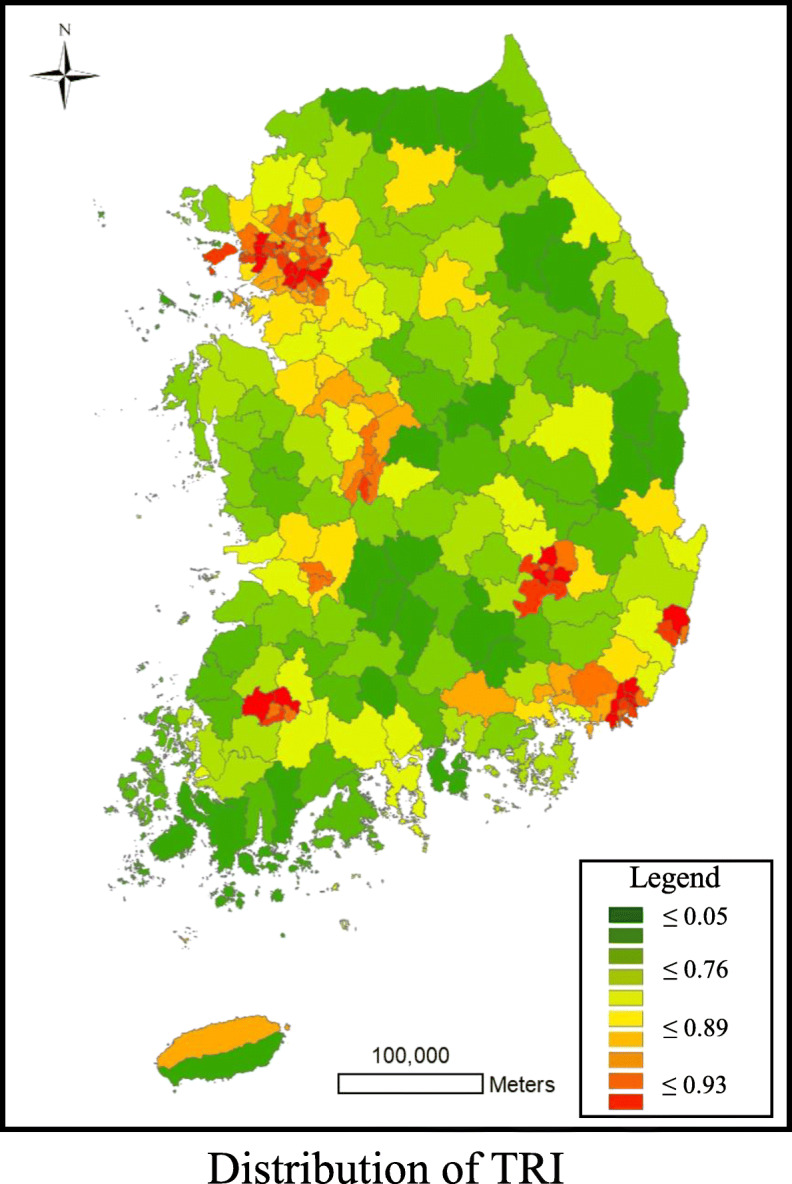
Geographical pattern of TRI

Table 4 and Fig. 4 show TRI’s descriptive statistics and geographical patterns (aggregated into municipal areal units). Correlation coefficients were calculated to compare the similarities between accessibility scores and the TRI index. Table 5 shows the correlation coefficients between the four metrics and the TRI values. We reported Spearman’s correlation coefficient and the Kendall-tau correlation coefficient, which are non-parametric versions of the Pearson correlation coefficient, because the units of measurement are different. As shown in Table 5, the proposed method shows the values 0.850 and 0.648 as the highest among the four metrics.^6^

**Table 4:** Descriptive statistics of TRI

Metric	Average (Standard deviation)	Maximum/Minimum	1st and 3rd Quartile (IQR)
TRI	0.65 (0.34)	0.96/0.00	0.39/0.92 (0.52)

**Table 5:** Correlation between the TRI and the four metrics

Correlation Coefficient	2SFCA	3SFCA	M2SFCA	clmFCA
Spearman	0.675	0.730	0.703	0.850
Kendall-tau	0.487	0.559	0.517	0.648

## Discussion

This study proposes a new accessibility measure type that explicitly incorporates the patients’ willingness to use a healthcare provider. A realistic and plausible metric to measure access to health services is the first and most crucial step in designing better policies to improve accessibility. However, obtaining real observations requires tremendous costs and effort. It is sometimes impossible to gather such observations because of practical limitations such as difficulty accessing private information. Conversely, the use of traditional metrics to calculate potential accessibility has a drawback. Most prior measures do not consider realistic demand preferences. Therefore, the results of these methods may provide incorrect information to policymakers. This may lead policymakers to design policies that are sub-optimal for the population. Motivated by this, the present study designs a new FCA metric that incorporates a discrete choice model. To the best of the author’s knowledge, the proposed metric involving a rigorous mathematical model explaining individuals’ willingness to choose a specific healthcare provider has not been studied in related literature. Therefore, it takes up a unique position in related areas of study.

As mentioned earlier, the advantage of using the clmFCA is its ability to more realistically identify potential demand for each healthcare provider. Specifically, the clmFCA considers patients’ realistic behavior, such as their bypass behavior, by incorporating the hospital selection. Bypass behavior generally affects the size of potential demand in two different ways [31, 32]. For undesirable healthcare providers, overestimations are avoided to a possible extent. Additionally, it also helps in correcting underestimations for attractive healthcare providers by considering unexpected arrivals to these providers. In particular, patients being able to freely choose their hospital based on the individual’s preference is a typical situation in Korea. These preferences include the patient’s travel time to the hospital.

It must be noted that most FCA metrics use simple procedures in estimating the potential demand for services. The 2SFCA assumes that the entire population has equal opportunities to use service providers within their catchment areas. On the other hand, the 3SFCA considers the competition effect when estimating demand. However, it simply divides the proportion of weight calculated by travel time. Moreover, M2FCA and E2SFCA primarily use a decay function based on travel time. Several FCA-type metrics consider non-spatial factors (e.g., congestion in hospitals and transportation types). Nonetheless, they considered non-spatial factors in a simple (or rough) manner. The present study is novel because it explicitly combines a discrete choice model with FCA measures. In doing so, this study provides a reasonable way to answer the question of how an individual selects a hospital. Thus, this contributes to obtaining more accurate and realistic results regarding accessibility to health services.

Another contribution of this study is its applicability. A general method of calculating potential accessibility, applied to various healthcare systems, is proposed here by incorporating a discrete choice model into an accessibility model. Such a method can adopt the unique characteristics of healthcare systems in the first step of the clmFCA. Traditional FCA metrics cannot consider the unique characteristics of various health systems because they do not account for the characteristics of different healthcare systems. However, the proposed method appropriately reflects such characteristics using a discrete choice model. Therefore, this would be one of the proposed model’s methodological merits as compared to the classical approaches.

The effectiveness of the clmFCA can be twofold. First, it hedges accessibility’s overestimation. Based on the accessibility scores, the clmFCA primarily avoids overestimating the potential accessibility to healthcare providers. This is magnified when compared with the results of the 2SFCA. The 2SFCA often provides unrealistic (i.e., overestimated) access values because it does not have an explicitly designed demand estimation model. Particularly, accessibility in regions where populations overlap in choosing several providers (e.g., metropolitan or urban areas) is more likely to be overestimated because it does not consider patients’ realistic preferences. However, the clmFCA accounts for this characteristic. That is, the proposed metric can provide realistic accessibility measures through a well-estimated demand model.

Second, the results from the clmFCA match well with the revealed accessibility. Table 5 presents the results, where it can be seen that the clmFCA provides accessibility trends similar to the TRI index. This is confirmed by analyzing the correlation coefficient between FCA-type metrics and the TRI index. In the clmFCA, the correlation coefficient’s value is greater than 0.80. Such a value implies a strong correlation between the potential accessibility from the clmFCA and the TRI index. In other words, the proposed method calculates appropriate values of accessibility where it should be. This would be beneficial for policymakers who design policies and strategies to improve healthcare delivery systems. For example, policymakers can design a way of deploying resources to medically serve underserved areas. They may likewise provide tailored packages of policies using the results from the clmFCA to cover demand considering their living areas.

Although this study proposes a new type of accessibility measure, it has several limitations. One limitation is that a choice model is initially required to execute the clmFCA. If realistic data on patients’ willingness to visit hospitals are not available, this study may not be superior to the existing methods. Another limitation is the need for access to more relevant data to calculate healthcare access. Although this study adapts patients’ preference in calculating healthcare access, it cannot use the finest level of demand (i.e., each patient’s residential location) because of practical challenges such as access to private and confidential information. Moreover, the limited access to information on patient characteristics can act as a barrier to obtaining a more realistic patient behavior estimation model.

## Conclusion

This study addresses the problem of calculating access to healthcare providers by designing a new FCA metric. It provides an access measure that realistically identifies patients’ accessibility to healthcare by incorporating their willingness to use a healthcare provider into the clmFCA. The study’s technical contribution is the development of a new metric that can capture a patient’s willingness to choose a hospital, leading to a more realistic potential accessibility measure. Such a metric has not been studied in related fields to the best of the author’s knowledge. Next, this study applies the clmFCA to real-world cases using nationwide data for obstetric care in Korea. The experimental results revealed the advantage of using the clmFCA. That is, it can estimate the potential demand for services as accurately as possible. Nonetheless, several areas can be further addressed. Employing more sophisticated choice models (e.g., the health belief model) or adopting rigorous mathematical models (including machine learning methods to predict potential demand for health services) can be considered in future studies.

## Supplementary Information

**Additional file 1.**

## Data Availability

The data analyzed in this study are from the Korean government, and the data analysis is ongoing for subsequent publications. Therefore, the datasets are not publicly available but are available on request from the corresponding author.

## Abbreviations

CLM: Conditional logit model
clmFCA: Conditional logit model-based floating catchment area
FCA: Floating catchment area
M2SFCA: Modified two step floating catchment area
3SFCA: Three-step floating catchment area
2SFCA: Two-step floating catchment area
TRI: Timely relevance index

## Footnotes

1 It is also worth noting that because the choice probability formula of the CLM is closed-form and only contains measurable factors, it can be easily calculated. That is, it can incorporate various factors that explain the choice process of the population as long as the utility function has a computable form.

2 McFadden’s pseudo R-squared (0.2748) and variable inflation factor for all variables (≤ 1.2) confirm that this model is statistically valid. The authors also conducted a five-fold cross-validation process and confirmed that it is acceptable to use the model for predictions (R^2^ between the actual usage and the predicted numbers of visits attains 0.7195 on average). It is worth noting that their model has been verified by several experts, including physicians, and the overall results and tendencies are consistent with their practical application.

3 In this study, obtaining personal data is impossible due to privacy issues, as demographic information along with residential location information and hospital visit records may identify specific individuals. If available, it seems reasonable that a finder segmentation of the fertile group may provide a better model to describe the hospital choice behavior by each segment.

4 A 60-min threshold is based on the results of the nationwide study in which the author was involved [26]. In this study, a threshold is determined by considering both expert’s opinions and prior related studies. It is worth noting that this is a parameter that can vary when it applies to different problems.

5 Considering that obtaining revealed accessibility requires tremendous effort and resources, we believe that TRI is appropriate to use as a proxy for revealed accessibility. It is worth noting that the goal of our study is to design more “realistic” potential accessibility; it is thus reasonable to compare the results of our measure with TRI.

6 We also reported the ranking of accessibility scores of the four accessibility metrics and the TRI index to Section 1.6 of the Supplemental Information.
