# Supplementary material for: A model for measuring healthcare accessibility using the behavior of demand: a conditional logit model-based floating catchment area method
Source: BMC Health Serv Res. 2021 Jul 6;21:660. doi: 10.1186/s12913-021-06654-3 (PMC8259122; doi:10.1186/s12913-021-06654-3)
Supplement: Supplementary file 1 — Additional file 1. [file 12913_2021_6654_MOESM1_ESM.docx]

**Supplemental**

- 1. **Benchmarking FCA metrics**

In this section, we briefly review the benchmarking FCA measures. To provide an elaborate explanation of the measures, we introduce the indices applied in the study. Let us define *i* ∈ I = {1, 2, 3, …, $\bar{i}$} as the locations of demand (population or patients), and *j* ∈ J = {1, 2, 3, …, $\bar{j}$} as the locations of suppliers (hospitals). We also define the boundary of the catchment area. Let *d_ij_* be the travel time from the demand location *i* to the hospital location *j*, and let *d^*^* be the threshold of travel time, which determines the geographical boundary of the catchment area. Then, if the *d_ij_*, is smaller than *d^*^*, the demand location *i* is covered by the hospital *j*. Using this property, we can define I_j_ = {*i* | *i* ∈ I, and *d_ij_* ≤ *d^*^* for fixed *j*} to be the set of demand locations covered by the supply site *j*. Likewise, let J_i_ = {*j* | *j* ∈ J, and *d_ij_* ≤ *d^*^* for fixed *i*} be the set of locations of supply sites which patients can reach from the demand location *i*. For the latter, let *D_i_* be the population living in the location *i*, and let *S_j_* be the capacity of the hospital *j*.

**1.1.1 2SFCA**

The 2SFCA is the pioneering metric in the FCA family. Motivated by the gravity model, it calculates accessibility for each demand location *i* in two steps. First, the supply to demand ratio for each facility is computed. Here, supply is typically considered as the number of beds (or physicians) in each hospital, and the demand for this calculation is included in the catchment area of *j*. Next, we sum up this ratio for each demand location *i*. The detailed algorithm is presented in Algorithm 1.

Algorithm 1. The 2SFCA algorithm

| **Input**{*S_j_* for *j* ∈ J, *D_i_* for *i* ∈ I} = **Output**{A_i_, for *i* ∈ I} |
| --- |
| **Step 1**. Calculate the supply to demand ratio, *R_j_*, for each facility *j*   - $R_{j}= \frac{S_{j}}{\sum_{i\in I_{j}} D_{i}}$   **Step 2**. Calculate the spatial accessibility, *A_i_*, by summing the ratio, *R_j_*   - $A_{i}= \sum_{j\in J_{i}} R_{j}$ |

**1.1.2 3SFCA**

Though the 2SFCA was effective in measuring accessibility in many areas, it crucially failed to address one realistic feature—potential competition among the population. In other words, the 2SFCA equally allocates demand to hospitals without considering the availability of other nearby hospitals. Therefore, it overestimates the demands on a hospital. Considering that overestimating demand will result in an unreliable access score for decision makers, this should be properly addressed.

The 3SFCA was developed to overcome the shortcomings in the 2SFCA. It proposed a new metric which assumes that the demand for hospitals is adjusted by the availability of hospitals. Specifically, by introducing a competition weight (*G*) which is based on travel time, it calculates a reasonable amount of demand, and thereby reduces the risk of demand overestimation. Many studies with virtual/real-world experiments have confirmed the usefulness of the 3SFCA over prior measures, including the 2SFCA. Algorithm 2 shows the detailed procedure of computing the 3SFCA score.

Algorithm 2. The 3SFCA algorithm

| **Input**{*W_ij_* for *i* ∈ I, *j* ∈ J, *S_j_* for *j* ∈ J, *D_i_* for *i* ∈ I}= **Output**{A_i_, for *i* ∈ I} |
| --- |
| **Step 1**. Calculate the selection weight, *G_ij_*_,_ for each demand–supply pair (*i*, *j*)   - $G_{ij}= \frac{W_{ij}}{\sum_{j\in J_{i}} W_{j}}$, where *W_ij_* is the travel impedance from *i* to *j*, $W_{j}= \sum_{i\in I_{j}} W_{ij}$   **Step 2**. Calculate the supply to weighted demand ratio, *R_j_*, for each facility *j*   - $R_{j}= \frac{S_{j}}{\sum_{i\in I_{j}} D_{i}W_{ij}G_{ij}}$   **Step 3**. Calculate the spatial accessibility, *A_i_*_,_ by the multiplication of *R_j_*, *W_ij_*, and *G_ij_*   - $A_{i}= \sum_{j\in J_{i}} R_{j}W_{ij}G_{ij}$ |

As seen in Algorithm 2, one notable point is that the 3SFCA includes one more step than the 2SFCA. Specifically, in step 1, the population’s hospital selection weight is calculated, and this information is used to appropriately allocate demand to hospitals. However, it should be noted that the 3SFCA simply uses travel time cost as a selection preference.

**1.1.3 M2SFCA**

The last benchmarking measure is the M2SFCA proposed by Delamater (2013). It is developed from the motivation that all prior metrics have the same underlying assumption—all suppliers’ locations are optimally configured to meet the needs for health services. In other words, the prior metrics intrinsically assume that the system is a single container, and all opportunities to use the services are fully allocated, regardless of the configuration of the system.

| 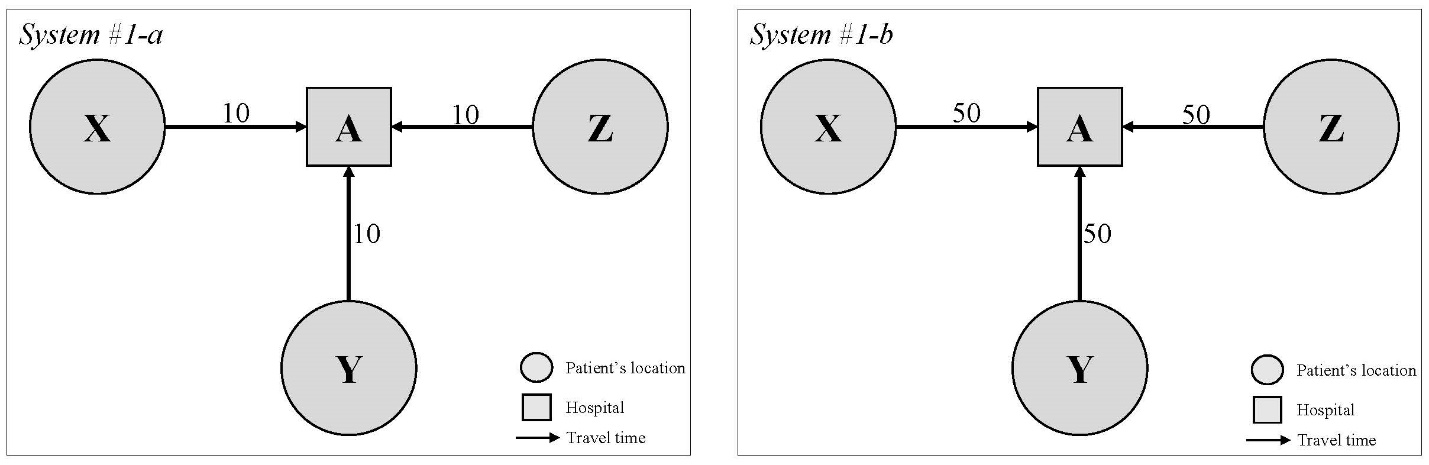 |
| --- |

Figure 1 Simulated systems which represent different configurations of service facilities

For instance, let us suppose that a system has three demand locations (X, Y, and Z) and one hospital (A). Each of the demand locations has a population of 100, and hospital A has 10 beds (Figure 1). Numbers on each arrow indicate the travel time between demand and supply. Then, all prior FCA metrics provide the same accessibility score by assuming that all opportunities (10 beds) will be shared by a population of 300. Therefore, the total opportunities will be the same regardless of how the system is configured. However, in real-world settings, system #1-a and system #1-b have very different accessibility. Demand in system #1-a has more opportunities than the population in system #1-b. Delamater (2013) pointed this out in his paper, and proposed a new metric, the M2SFCA, to overcome this limitation. As shown in Figure 1, the M2SFCA captures the suboptimal configuration of the suppliers’ network structure.

Specifically, to appropriately account for this problem, Delamater (2013) presented the M2SFCA, which works well even in a suboptimal configuration of supplier’s locations. Overall, the M2SFCA is similar to the prior metric, the E2SFCA; however, the difference is that the M2SFCA considers the decaying function to the supply side (See step 1 in Algorithm 3). By doing so, it offers more realistic access levels where the existing models do not.

Algorithm 3. The M2SFCA algorithm

| **Input**{*W_ij_* for *i* ∈ I, *j* ∈ J, *S_j_* for *j* ∈ J, *D_i_* for *i* ∈ I}= **Output**{A_i_, for *i* ∈ I} |
| --- |
| **Step 1**. Calculate the weighted supply to weighted demand ratio, *R_j_*, for each facility *j*   - $R_{ij}= \frac{S_{j}W_{ij}}{\sum_{i\in I_{j}} D_{i}W_{ij}}$   **Step 2**. Calculate the spatial accessibility, *A_i_*_,_ by the multiplication of *R_j_*, *W_ij_*   - $A_{i}= \sum_{j\in J_{i}} R_{ij}W_{ij}$ |

To sum up, distinguishable characteristics of each of the three benchmarking methods can be listed as follows:

Table 1 Distinguishable characteristics of three benchmarking methods

| **Feature** | **2SFCA** | **3SFCA** | **M2SFCA** | **clmFCA** |
| --- | --- | --- | --- | --- |
| Unit of measure | The ratio of the supply to demand | | | |
| Impedance of travel time from demand *i*  to supply *j* | X | O | O | O |
| Competition between demands | X | O | X | O |
| Configuration of healthcare providers | X | X | O | O |

- 1. **Virtual experiments**

In this section, the advantages of the clmFCA over the three prior benchmarking measures were examined using virtual experiments. For this purpose, three measures – 2SFCA, 3SFCA, and M2SFCA were arbitrarily chosen.

To conduct virtual experiments, two different systems, which consist of several demand and supply locations were considered. The first system (system #2 in Figure 2) is considered to examine the bypass effect^[[1]](#footnote-1)^ and the second system (system #3 in Figure 2) is designed to verify the heterogeneous property of demand behavior. Circles (X, Y, and Z) in simulated systems represent locations of demand (e.g., patients), and squares (A and B) mean locations of supply (e.g., hospital). This experiment assumes that each demand location has a population of 100 and each hospital has 10 beds. Numbers above the black lines indicate the travel time (*min*) from the demand location to the supply site. For this virtual experiment, it is arbitrarily considered the Gaussian decay function (*w*) as an impedance function (*w* = *f*(*d*) = *exp*(*-d^2^/1000*)), and the catchment area threshold is also arbitrarily determined as 60 minutes. Additionally, the value of *P_ij_* to compute accessibility from the clmFCA (Table 1) is assigned to magnify patients’ bypass behavior or show heterogenetic properties in selecting hospitals.

| 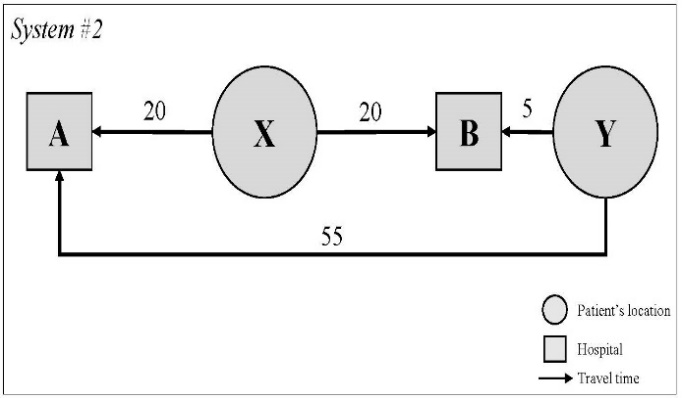 | 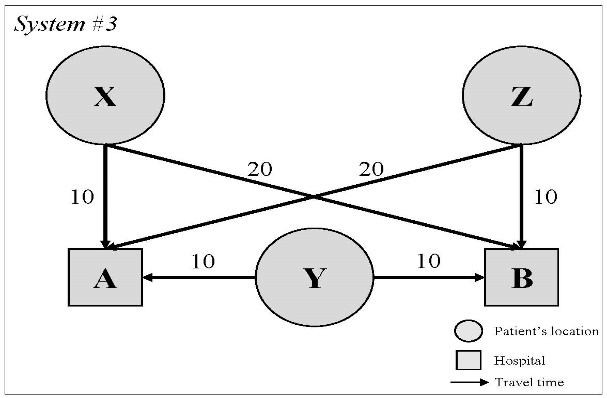 |
| --- | --- |
|  | |

Figure 2 Simulated systems to verify the advantages of the clmFCA

Table 2 P_ij_ for three simulated systems

| **System** | **Demand** | **A** | **B** |
| --- | --- | --- | --- |
| System #2 | X | 0.1 | 0.9 |
|  | Y | 0.0 | 1.0 |
| System #3 | X | 0.7 | 0.3 |
|  | Y | 0.5 | 0.5 |
|  | Z | 0.4 | 0.6 |

Table 2 presents the accessibility scores of the three simulated systems. The advantages of clmFCA can be observed from different angles. Let us first examine system #2. The three prior metrics provide similar results for system #2. In the case of the M2SFCA, though it does not dramatically alter the value of accessibility, accessibility is discounted due to travel time to A. This is because the accessibility value from the M2SFCA is lower than that from the first two prior measures (2SFCA and 3SFCA). However, the outcome of the clmFCA is very different from that of the prior measures. Specifically, according to the given values of choice probability in Table 2, only 10% of the population at X will visit hospital A, and no populations in Y will use this hospital. Therefore, this 10% population at X will not experience any competition when using healthcare services, which guarantees that the accessibility of X will be 0.1 or above. However, 90% of the population at X and all population at Y will visit hospital B, which incurs high competition when a patient chooses hospital B. Therefore, in the view of the population at Y, accessibility to health services will be around 0.05. The clmFCA can reflect such properties well enough compared to the benchmarking methods. The detailed calculation process is shown in Table 4.

Table 3 Accessibility from the benchmarking metrics and the clmFCA

| **System** | **Demand** | **2SFCA** | **3SFCA** | **M2SFCA** | **clmFCA** |
| --- | --- | --- | --- | --- | --- |
| System #2 | X | 0.100 | 0.131 | 0.090 | 0.147 |
|  | Y | 0.100 | 0.069 | 0.058 | 0.053 |
| System #3 | X | 0.067 | 0.061 | 0.051 | 0.065 |
|  | Y | 0.067 | 0.079 | 0.066 | 0.067 |
|  | Z | 0.067 | 0.061 | 0.051 | 0.068 |

In case of system #3, another advantage of the clmFCA can be found. Specifically, the populations at X and Z in system #3 seem to have an equivalent stance. They have two suppliers, and the travel time to each supplier is the same as well. Because of this configuration, all prior measures produce equal accessibility scores. However, in reality, the *P_ij_* of X and Z are different. The clmFCA therefore provides different accessibility scores for X and Z.

To sum up, virtual experiments successfully reveal the effectiveness of the proposed method. Specifically, the clmFCA can appropriately consider two patients’ realistic behaviors (bypass behavior and heterogeneous nature of the population) in computing access to health services.

Table 4 Details on the accessibility calculation process (system #2)

| System #2 | Calculation Process |
| --- | --- |
| 2SFCA | R_A_ = $\frac{10}{200}$ = 0.05  R_B_ = $\frac{10}{200}$ = 0.05  A_X_ = R_A_ + R_B_ = $\frac{10}{200}$ + $\frac{10}{200}$ = 0.1  A_Y_ = R_A_ + R_B_ = $\frac{10}{200}$ + $\frac{10}{200}$ = 0.1 |
| 3SFCA | G_XA_ = $\frac{0.67}{0.67+0.049}$ = 0.932  G_XB_ = $\frac{0.67}{0.67+0.975}$ = 0.407  G_YA_ = $\frac{0.049}{0.67+0.049}$ = 0.068  G_YB_ = $\frac{0.975}{0.67+0.975}$ = 0.593  R_A_ = $\frac{10}{100\times0.670\times0.932+100 \times0.049\times0.068}$ = 0.159  R_B_ = $\frac{10}{100\times0.670\times0.407+100 \times0.975\times0.593}$ = 0.118  A_X_ = 0.159 x 0.670 x 0.932 + 0.118 x 0.670 x 0.407 = 0.131  A_Y_ = 0.159 x 0.049 x 0.068 + 0.118 x 0.975 x 0.593 = 0.069 |
| M2SFCA | R_XA_ = $\frac{10\times0.670}{100\times0.670+100 \times0.049}$ = 0.093  R_XB_ =$\frac{10\times0.67}{100\times0.67+100\times0.975}$ = 0.041  R_YA_ =$\frac{10\times0.049}{100\times0.049+100\times0.67}$ = 0.007  R_YB_ =$\frac{10\times0.975}{100\times0.975+100 \times0.67}$ = 0.059  A_X_ = 0.093 x 0.670 + 0.041 x 0.670 = 0.090  A_Y_ = 0.007 x 0.049 + 0.059 x 0.975 = 0.058 |
| clmFCA | R_XA_ = $\frac{10}{100\times0.1+100 \times0.0}$ = 1  R_XB_ =$\frac{10}{100\times0.9+100\times1.0}$ = 0.053  R_YA_ =$\frac{10}{100\times0.1+100\times0.0}$ = 1  R_YB_ =$\frac{10}{100\times0.9+100 \times1.0}$ = 0.053  A_X_ = 1 x 0.1 + 0.053 x 0.9 = 0.147  A_Y_ = 1 x 0.0 + 0.053 x 0.1 = 0.053 |

- 1. **The choice model used in this study**

**1.3.1 Data preparation**

In the choice model used in this study by Hwang et al. [30], the variables constructing the observable utility, V_ij_,were selected, level of the hospital, urbanization of the hospital location, number of obstetrics specialists, and travel time. For this, they reviewed articles including Victoor’s work^[[2]](#footnote-2)^. They categorized factors affecting hospital choice behavior. The final variables were chosen considering the availability of data.

In Korea, the level of the hospital would be a key factor when selecting hospitals. Except for the high-risk pregnant women, most pregnant women prefer low-level hospital. Urbanization of the hospital’s location is also a key factor. In Korea, many people use hospitals located in the downtown area due to many reasons. The number of obstetrics specialists and the size of the hospital are crucial factors. According to Bronstein’s work^[[3]](#footnote-3)^, the number of doctors in the office is an important factor in selecting a hospital. Travel time is a typical factor that affects the choice of hospital.

However, to construct studied data, Hwang et al. [30] used three sources: 1) medical records in use – A birth data in 2016 in Korea collected by the National Health Insurance Service. It contains the residential information of pregnant women and the location of the hospital. 2) Information on each hospital, which includes facilities, medical equipment, and workforce information. This is provided by the Health Insurance Review and Assessment service. Lastly, 3) travel time between residential areal units to a hospital is calculated by the GIS analysis provided by the National Transportation Database Center. By excluding some data that does not match the criteria for the analysis, Hwang et al. [30] finally confirmed 389,732 cases (from 399,096 cases).

On the other hand, the statistical results of the choice model is presented below:

Table 5 Results of the patients' hospital choice model (Hwang et al. [30])

| **Factor** | **Estimate** | **Odds ratio** | **Standard error** | **p-value** |
| --- | --- | --- | --- | --- |
| Hospital level  LvL (reference)  LvH  Hospital location  Urb_Metro_ (reference)  Urb_City_  Urb_Rural_  Number of obstetricians  Travel time (min.) | -1.0722  -0.1738  -0.8619  0.1511  -0.0637 | 0.34  0.84  0.42  1.16  0.94 | 0.0287  0.0305  0.0499  0.0023  0.0001 | < 0.0001  < 0.0001  < 0.0001  < 0.0001  < 0.0001 |

Next, the following validation processes were conducted. First, they have checked the difference between the specific obstetric care unit’s actual visit ratio and the choice probability of the care unit of each residential areal unit. Then, compared with the benchmarking rule (distance-based patient assignment rule), they have evaluated the root mean squared error (RMSE). As presented in Table 6, the RMSE of the proposed model has shown values lower than that of the benchmarking method.

Table 6 RMSE and Accuracy of the proposed model

|  | The proposed model | The distance-based rule |
| --- | --- | --- |
| RMSE (Average/Standard deviation) | 0.018 (0.016) | 0.020 (0.011) |
| Accuracy | 90.08% | 69.84% |

Secondly, they have calculated the expected visits using two approaches (the choice probability and the distance-based rule) and compared them with the actual visit records. The results indicate that the accuracy of the expected visits based on the choice probability was nearly 90%, whereas the distance-based rules were around 70%. Considering a sort of processes, we believe that the choice model has enough predictive power, leading to the proposed model’s validity.

- 1. **Ranking of Accessibility Scores**

We reported the ranking of accessibility scores of the four metrics and TRI index in Table 7. By considering the results’ visibility, the ranking is presented by aggregating accessibility scores to a province level. As shown in Table 7, in TRI, Gangwon is the lowest, followed by Jeonnam and Gyeongbuk. In 2SFCA, Chungnam shows the lowest accessibility, which is followed by Gyeongbuk and Chungbuk. In the proposed method, Jeonnam has the lowest accessibility score, followed by Gangwon and Gyeongbuk.

Table 7 Ranking of accessibility scores (in Province level)

| Rank | TRI | 2SFCA | 3SFCA | M2SFCA | clmFCA |
| --- | --- | --- | --- | --- | --- |
| 1 | Gangwon | Chungnam | Chungbuk | Sejong | Jeonnam |
| 2 | Jeonnam | Gyeongbuk | Jeonnam | Chungbuk | Gangwon |
| 3 | Gyeongbuk | Chungbuk | Gyeongbuk | Gyeongbuk | Gyeongbuk |
| 4 | Jeonbuk | Ulsan | Chungnam | Jeonnam | Chungbuk |
| 5 | Jeju | Gangwon | Jeonbuk | Chungnam | Jeonbuk |
| 6 | Gyeongnam | Gyeongnam | Gyeongnam | Gyeongnam | Jeju |
| 7 | Chungnam | Jeonnam | Gangwon | Gyeonggi | Gyeongnam |
| 8 | Chungbuk | Jeonbuk | Ulsan | Jeonbuk | Chungnam |
| 9 | Incheon | Incheon | Jeju | Incheon | Ulsan |
| 10 | Sejong | Gyeonggi | Gyeonggi | Gangwon | Gwangju |
| 11 | Gyeonggi | Gwangju | Sejong | Ulsan | Sejong |
| 12 | Ulsan | Daejeon | Incheon | Jeju | Daegu |
| 13 | Seoul | Daegu | Daegu | Seoul | Daejeon |
| 14 | Daejeon | Jeju | Gwangju | Busan | Busan |
| 15 | Gwangju | Busan | Daejeon | Daegu | Incheon |
| 16 | Busan | Seoul | Seoul | Gwangju | Gyeonggi |
| 17 | Daegu | Sejong | Busan | Daejeon | Seoul |

Overall, the proposed method’s rankings are similar to the ones of TRI, compared to the other metrics. Particularly, it matches well with the first to eighth-ranked provinces having lower accessibility scores to healthcare providers. This would be beneficial for policymakers who design policies and strategies to make better healthcare delivery systems. For example, policymakers can design a way of deploying resources to serve medically underserved areas^[[4]](#footnote-4)^ or providing tailored packages of policies to cover demand considering their living area using the results from the clmFCA.

1. As noted in earlier studies, bypassing frequently occurs in the use of health services [31, 32]. In particular, it is a very typical phenomenon in Korea where patients can freely choose their hospital based on the individual’s preference, including travel time to hospital [↑](#footnote-ref-1)
2. Victoor A et al. Determinant of patient choice of healthcare providers: a scoping review. BMC Health Serv Res. 2012;12:272. [↑](#footnote-ref-2)
3. Bornstein H et al. Choosing a doctor: an exploratory study of factors influencing patients’ choice of a primary care doctor. J Eval Clin Pract. 2000; 6:255-62. [↑](#footnote-ref-3)
4. H Jang et al., Designing robust rollout plan for better rural perinatal care system in Korea. Eur J Oper Res. 2019;274: 730-42 [↑](#footnote-ref-4)
